# An Analysis of the Different Salt-Tolerance Mechanisms in Rice Cultivars Induced by Cerium Oxide Nanoparticles

**DOI:** 10.3390/antiox14080994

**Published:** 2025-08-13

**Authors:** Chunmei Yang, Qing Bu, Tao Su, Tian Wang, Zaid Khan, Mingwei Li, Juntian Wu, Xiaodan Di, Yong Chen, Jing An

**Affiliations:** 1 College of Agriculture, South China Agricultural University, Guangzhou 510642, China; 2 College of Natural Resources and Environment, South China Agricultural University, Guangzhou 510642, China

**Keywords:** PNC priming, salt stress, different rice cultivars, mechanism differences

## Abstract

Cerium oxide nanoparticles (CeO_2_NPs) can boost crops’ salt tolerance, yet their regulatory mechanisms in rice cultivars with contrasting salt tolerance remain unclear. This study investigated the regulatory differences in poly (acrylic acid)-coated nanoceria (PNC)-primed in salt-sensitive (Huanghuazhan, H) and salt-tolerant (Xiangliangyou900, X) rice. The results showed that PNC priming improved salt tolerance in two cultivars, but the underlying mechanisms differed. In the H cultivar, the enhanced tolerance was primarily attributed to enhanced photosynthesis (net photosynthesis and transpiration rates were 53.27% and 20.52% higher than the X cultivar); increased abscisic acid (ABA) content (up by 18.80% compared to the X cultivar), and activated stress-responsive signaling. Metabolomics further revealed that the differential metabolites were enriched in galactose metabolism, ascorbate, and aldarate metabolism, synergistically maintaining intracellular redox balance. In the X cultivar, PNC boosted reactive oxygen species’ (ROS) scavenging capacity (catalase (CAT) increased 36.07%, H_2_O_2_ and malondialdehyde (MDA) decreased 27.31% and 48.61% compared to H); elevated endogenous indole-3-acetic acid (IAA) and gibberellic acid3 (GA3) levels by 9.55% and 9.08%; and specifically activated cellular defense response and glutathione metabolism. Transcriptome analysis further revealed that the expression of IAA/GA3 signal-responsive genes *(OsARGOS*/*OsGASR2*) and antioxidant genes (*OsCatA*, *OsAPX1*) were significantly higher in the X cultivar than the H cultivar (*p* < 0.05), whereas the H cultivar showed higher expression of GST and ABA-related genes. This study provides a new perspective for the mechanism of PNC-enhanced salt tolerance in rice.

## 1. Introduction

Soil salinity has become an environmental problem globally due to climate change, fertilizer and pesticide misuse, and irrational irrigation by humans, affecting plant growth and development [1]. More than 1 billion km^2^ of land was affected by salt stress in the world; China has about 9.9 × 10^7^ km^2^ of saline land, which is the third largest quantity in the world. This seriously threatens the sustainable development of agricultural production and the ecological environment [2]. The selection and breeding of salt-tolerant cultivars and enhancement of crop salt tolerance are key measures to solve the problem of soil salinity, which is of great significance in guaranteeing food security and promoting sustainable development of agriculture.

Rice is the staple food for nearly half of the world’s population and is the main food crop in South China. However, different rice cultivars have varying degrees of salt tolerance and distinct response and adaptation strategies to salt stress [3]. Under salt stress, some cultivars maintain osmotic balance by accumulating osmoregulatory substances [4]; some maintain ionic balance by restricting Na^+^ and Cl^−^ uptake and transport [5]; and some cultivars improve the antioxidant defense of the plant by regulating antioxidant enzyme activities [6]. Moreover, it has been shown that under salt stress, salt-tolerant cultivars had higher antioxidant enzyme activities, proline, and H_2_O_2_ content; sensitive cultivars had higher MDA and chlorophyll content [7]. This indicates that the salt-tolerance mechanisms vary among rice cultivars. Therefore, understanding the salt-tolerance mechanisms of different cultivars is of great theoretical significance for improving the utilization efficiency of saline soil.

CeO_2_NPs is an emerging nanomaterial with excellent antioxidant properties that can scavenge free radicals in plants [8], thus reducing the damage of biotic and abiotic stresses in plants. Moreover, CeO_2_NPs can improve plant resistance by promoting plant growth and development [9,10]. Previous studies have shown that CeO_2_NPs improves salt tolerance in oilseed rape [11], cucumber [12], cotton [13], rice [14], and other crops. The mechanisms were mainly related to ROS scavenging [15], ion homeostasis [16,17], and α-amylase activity [18]. However, these studies mainly focused on single-crop cultivars. There is lack of systematic and in-depth research on the response and adaptation mechanisms of diverse cultivars to salt damage when treated with CeO_2_NPs and the relationship between these mechanisms and salt tolerance.

Seed germination is the first step in plant growth and development, and seed priming is one of the most widely used techniques to enhance seed germination under adverse conditions [11]. Compared with foliar spraying, PNC priming can be absorbed by seeds at low doses, avoiding the risk of cerium residue that may be caused by high-dose spraying; moreover, the seed stage is a critical window for establishment of ROS homeostasis and hormone signaling; PNC priming can activate antioxidant signals at an early stage, enabling plants to mount a rapid defensive response when salt stress occurs. Therefore, in this study, rice seeds were initiated with PNC to investigate the differential salt-tolerance mechanisms of two rice cultivars (salt-sensitive H and salt-tolerant X) under salt stress. Firstly, we investigated the impact of PNC priming on the growth of H and X under salt stress; secondly, the physiological aspect of ROS and hormone levels was explored; finally, transcriptomic and metabolomic analyses were conducted to reveal the differences and similarities in gene expression and metabolites between H and X rice cultivars. So, as to reveal the distinct salt-tolerance mechanism of PNC priming in different rice cultivars. Through this study, we expect to provide new insights and revelations for the genetic improvement of salt-tolerant rice cultivars and the effective utilization of saline soils.

## 2. Materials and Methods

### 2.1. Materials

Huanghuazhan (H) is an early-maturing cultivar of conventional rice, purchased from Hunan Golden Nongfeng Seed Co. LTD, Changsha, China; Xiangliangyou900 (X) is a medium–late maturing cultivar of hybrid rice, provided by the Rice Institute of Hunan Academy of Agricultural Sciences. These two cultivars are widely cultivated and highly popular in southern China. Under salt stress, Huanghuazhan exhibits salt sensitivity, while Xiangliangyou900 displays salt tolerance [19,20].

PNC was synthesized in our laboratory. Briefly, polyacrylic acid and cerium nitrate were dissolved in water and then mixed, and ammonia water was added to the mixture, which was stirred at room temperature for 24 h. After centrifugation, the supernatant was filtered and collected, and PNC was obtained [21].

### 2.2. Experimental Design

In this experiment, salt-sensitive rice cultivar H and salt-tolerant rice cultivar X were used as materials. H and X rice seeds of uniform size were selected, sterilized with 5% (*v*/*v*) NaClO for 15 min, rinsed with deionized water for three times, and the following treatment was carried out.

#### 2.2.1. Effect of Different Priming Agents on Rice Seedling Growth Under Salt Stress

Surface-sterilized rice seeds were primed with 100 mg L^−1^ PNC (previously optimized in lab, Figure S1), or 100 mg L^−1^ Ce(NO_3_)_3_ at 28 °C for 48 h under dark conditions, with water priming used as control. After seed priming, seeds were rinsed and placed in Petri dishes for germination at 30 °C/14 h light and 25 °C/10 h dark for 48 h. Germinated seedings were transplanted into 1/4 Kimura nutrient solution [22], with pH adjusted to 5.5–6.0, renewed every 3 days. At the two-leaf stage, seedlings were exposed to 50 mM NaCl (previously optimized in lab, Figure S1) solution, with a control group receiving no NaCl. After 7 days, rice seedlings were harvested for agronomic trait measurements, with three biological replicates per treatment and five plants measured per replicate. The treatment settings were as follows: water priming (H_2_O), Ce(NO_3_)_3_ priming (Ce(NO_3_)_3_), PNC priming (PNC), water priming with 50 mM NaCl added (H_2_O + NaCl), Ce(NO_3_)_3_ priming with 50 mM NaCl added (Ce(NO_3_)_3_ + NaCl), PNC priming with 50 mM NaCl added (PNC + NaCl).

#### 2.2.2. PNC Priming Effects on Rice Physiological and Biochemical Under Salt Stress

Seeds were primed for 48 h in 0 or 100 mg L^−1^ PNC, rinsed, and then germinated in an incubator for 48 h. Afterward, the seedlings were transferred to the nutrient solution for cultivation, and the cultivation conditions were consistent with those in Section 2.2.1. When the rice reached the two-leaf stage, they were treated with 50 mM NaCl solution, with a control group receiving no NaCl. On the 7th day of salt stress, the photosynthetic parameters of the second set of fully expanded leaves were measured using a plant photosynthesis meter. Roots were then harvested, one portion was immediately stained with DAB and NBT for ROS visualization, another was stored at −80 °C after being flash-frozen in liquid nitrogen for antioxidant enzyme assays and phytohormone analyses. The experimental treatments were water priming (H_2_O), PNC priming (PNC), water priming with 50 mM NaCl added (H_2_O + NaCl), PNC priming with 50 mM NaCl added (PNC + NaCl). Each treatment included three biological replicates.

#### 2.2.3. PNC Priming Effects on Rice Omics Under Salt Stress

The treatment settings and plant cultivation conditions were consistent with those described in Section 2.2.2. At 7 days of salt stress treatment, the rice roots were harvested, rapidly frozen in liquid nitrogen, and stored at −80 °C for transcriptomics and metabolomics analysis.

### 2.3. Measurement of Agronomic Traits in Rice Seeding

After 7 days of salt stress treatment, the plant height and root length of the rice seedlings were measured with a ruler, and the fresh weight of rice plants was measured with a precision balance.

### 2.4. Measurement of Photosynthetic Parameters

At 7 days of salt stress treatment, net photosynthetic rate, stomatal conductance, transpiration rate, and the intercellular CO_2_ concentration of the second leaves were measured using a plant photosynthesis meter (LI-6400XT, LI-COR, Lincoln, NE, USA) between 9:00 and 11:00 a.m. on a sunny day. Four leaves were measured per treatment.

### 2.5. DAB Staining of Rice-Root Tips

Taking the rice-root system treated with salt for 7 days, the root tip was cut off (approximately 4–5 cm), placed in phosphate buffer (50 mM, pH 7.5) containing 0.5 mg mL^−1^ 3,3′-diaminobenzidine (DAB) staining solution, and stored at 28 °C in the dark for more than 8 h. The staining solution was poured off and rinsed three times with phosphate buffer. Photographs were taken to observe changes in the root tips, and the staining intensity was quantified using ImageJ-Fiji (1.46r; Java 1.6.0_20). Each treatment was repeated three times [21].

### 2.6. NBT Staining of Rice-Root Tips

Rice roots after 7 days of treatment were taken, the root tip was cut off (approximately 4–5 cm), then placed in phosphate buffer (50 mM, pH 7.5) containing 0.5 mg mL^−1^ nitrotetrazolium blue chloride (NBT) staining solution, and stored at 28 °C in the dark for 3 h. Photographs were taken to observe changes in the root tips, and the staining intensity was quantified using ImageJ-Fiji (1.46r; Java 1.6.0_20). Each treatment was repeated three times.

### 2.7. Determination of MDA Content

MDA content was determined by the thiobarbituric acid (TAB) colorimetric method. Fresh samples were homogenized in ice-cold 0.05 mol L^−1^ phosphate buffer (pH 7.8), centrifuged, and then the supernatant was collected. The supernatant was added to a mixture containing 0.5% TAB and 5% trichloroacetic acid, shaken, and boiled for 30 min. After centrifugation, the absorbance values were measured at 532, 600, and 450 nm [23].

### 2.8. Determination of Antioxidant Enzyme Activity

Extraction of crude enzyme solution: a fresh sample was homogenized in ice-cold 0.05 mol L^−1^ phosphate buffer (pH 7.8), centrifuged, and the supernatant was collected as the crude enzyme extract.

SOD was assayed using the nitro-blue tetrazolium photoreduction method, with one enzyme activity unit defined as 50% inhibition of nitro-blue tetrazolium photoreduction. CAT was determined as the decline in absorbance at 240 nm due to the oxidation of H_2_O_2_, and a decrease of 0.01 per minute was defined as one unit of enzyme activity. The guaiacol oxidation method was used for POD, and its activity was determined by the increase in absorbance at 470 nm caused by the oxidation of guaiacol, and an increase of 0.01 per minute was defined as one unit of enzyme activity [24].

### 2.9. Determination of Endogenous Hormone

The plant IAA, ABA, GA3, and jasmonic acid (JA) content in rice-root systems were determined using a double-antibody sandwich enzyme-linked immunosorbent assay (ELISA) kit. Briefly, 0.2 g of root tissue was sampled from seeding exposed to salt stress for 7 days, and the extraction and ELISA procedures were performed strictly according to the manufacturer’s instructions (Jiangsu Enzyme Immunity Industry Co., Yancheng, China). Absorbance at 450 nm was measured with an enzyme-linked immunosorbent assay reader (SynergyH1, Bio-Tek Instruments, Winooski, VT, USA); the sample concentrations were calculated from the standard curve.

### 2.10. Transcriptome and Bioinformatics Analysis of Rice-Root Systems

Total RNA was extracted using TIANGEN RNA Plant Plus Reagent Kit (TIANGEN Biotech (Beijing) Co., Ltd., Beijing, China), and RNA concentration and integrity were measured using Agilent 2100 RNA Nano 6000 Assay Kit (Agilent Technologies, Santa Clara, CA, USA). cDNA library construction and sequencing were carried out by Guangzhou Weiyu Zhihe Technology Co. Ltd., Guangzhou, China.

The obtained gene-expression data were compared between groups, and |log_2_ Fold change| ≥ 1, and FDR < 0.05 were used as the screening threshold for the differentially expressed genes (DEGs). From this, specific genes associated with the conditions were identified, and then the biological significance of these specific genes was further analyzed.

### 2.11. Non-Targeted Metabonomic Analysis

Ground freeze-dried root samples to a powder using a mill. The samples were mixed with the extraction solution (methanol–acetonitrile–water = 2:2:1, *v*/*v*) by shaking. Ultrasonicated for 30 min in an ice-water bath, placed for 1 h, centrifuged. The supernatant was taken, blown dry with a nitrogen blowing instrument, redissolved with acetonitrile–water = 1:1 (*v*/*v*), centrifuged, and the supernatant was taken for analysis by LC-MS.

Chromatographic conditions: the column used was a Waters UPLC BEH Amide column, the mobile phase A was ultrapure water, and B was acetonitrile, with 0.04% acetic acid added to both. The gradient elution conditions were 0–1 min, 85% B; 1–12 min, 65% B; 12–12.1 min, 40% B; 12.1–15 min, 40% B; 15–15.1 min, 85% B; 15.1–20 min, 85% B; and the flow rate was 0.3 mL min^−1^. Mass spectrometry conditions: electrospray ionization source, source temperature 600 °C, ion-source voltage −4500 V or 5500 V; the air-curtain gas was 20 psi, while the atomization gas and auxiliary gas were both 60 psi. Scanning was performed using multiple-reaction monitoring.

After the metabolites were identified based on the metabolic public databases HMDB, Metlin, and the self-constructed database VGDB, the resulting data matrices were uploaded to the KEY platform for data preprocessing. The preprocessed data matrices were analyzed by PCA, PLS-DA, and OPLS-DA to differentiate the differential metabolites between groups. According to the variable importance in projection (VIP), fold change (FC), and t-test of OPLS-DA model, the differentially expressed metabolites (DEMs) were screened by the screening criteria of |log_2_FC| ≥ 1, *p*-value < 0.05, and VIP value ≥ 1.

### 2.12. Statistical Analysis

All results were presented as the mean ± standard errors (SE). Data were processed using SPSS 20 software using ANOVA and Duncan’s test for multiple comparisons of means between treatments (*p* < 0.05). Analytical data were plotted using OriginPro 2021.

## 3. Results

### 3.1. Effect of PNC Priming on the Growth of Two Rice Cultivars Under Salt Stress

**Figure 1:**
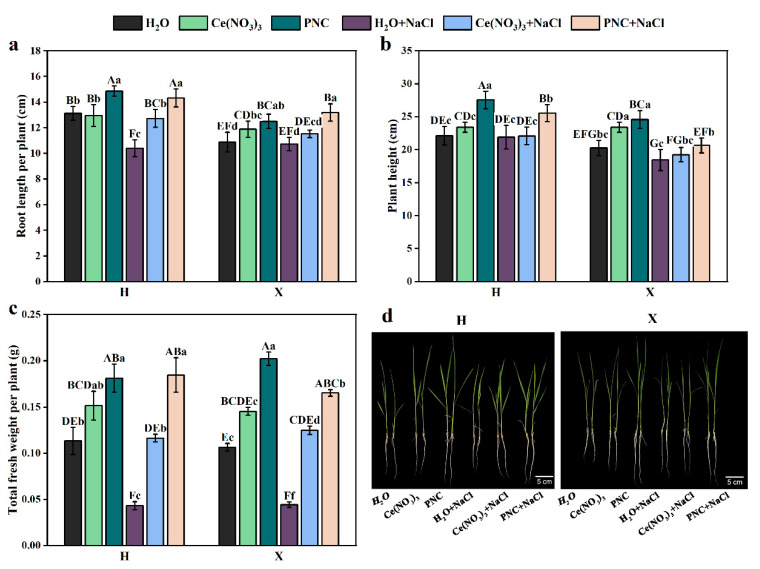
Effects of PNC priming on the growth of two rice cultivars under salt stress. (**a**) Root length after 7-day treatment; (**b**) plant height after 7-day treatment; (**c**) fresh weight after 7-day treatment; and (**d**) phenotype after 7-day treatment. Columns marked with different lowercase letters indicate significant differences among treatments within the same cultivars, while different uppercase letters denote significant differences between treatments of different cultivars, based on the Duncan test (*p* < 0.05, *n* = 5).

Under normal growth conditions, the rice leaves were bright green. The plant height, root length, and fresh weight of H and X both cultivars, primed with PNC, were significantly higher than those primed with water (Figure 1a–c). Under salt stress, rice leaves withered, and roots became thin and weak (Figure 1d). However, after PNC priming, the plant height, root length, and fresh weight of H and X cultivars were higher than those primed with H_2_O or Ce(NO_3_)_3_. Notably, PNC improved salt tolerance in the two rice cultivars differently. Under salt stress, PNC-primed cultivar H showed 23.74%, 8.65%, and 11.78% higher plant height, root length, and fresh weight than the X cultivar (Figure 1a–c). This indicated that PNC promoted rice growth and alleviated salt damage, with better phenotypic improvement in cultivar H than X.

### 3.2. Effect of PNC Priming on Photosynthesis in Two Rice Cultivars Under Salt Stress

**Figure 2:**
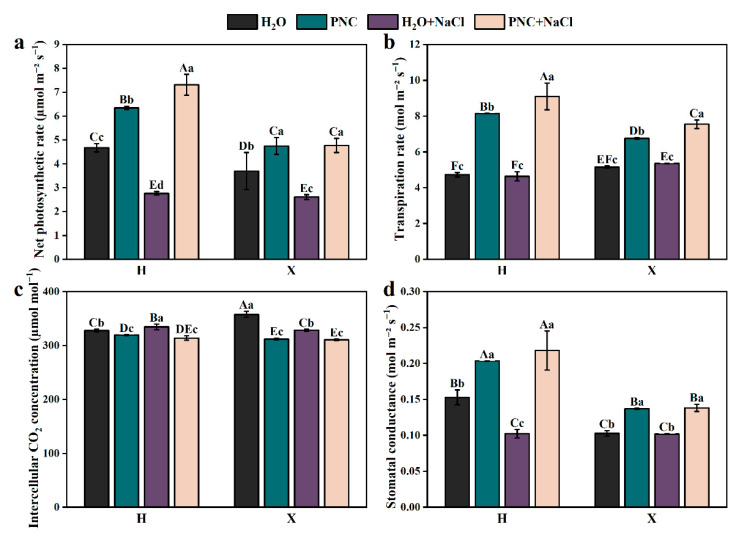
Effects of PNC priming on photosynthesis in two rice cultivars under salt stress. (**a**) Net photosynthetic rate; (**b**) transpiration rate; (**c**) intercellular CO_2_ concentration; and (**d**) stomatal conductance. Columns marked with different lowercase letters indicate significant differences among treatments within the same cultivars, while different uppercase letters denote significant differences between treatments of different cultivars, based on the Duncan test (*p* < 0.05, *n* = 4).

Under salt stress, the net photosynthetic rate of rice leaves declined sharply. However, PNC priming increased the net photosynthetic rate, transpiration rate, and stomatal conductance of cultivars H and X by 82.94–164.70, 41.05–96.22, and 35.57–113.59% (*p* < 0.05) (Figure 2a,b,d), respectively; while reducing intercellular CO_2_ by 5.40–6.15% (Figure 2c). Notably, under both normal and salt-stress conditions, PNC-primed cultivar H had a significantly higher net photosynthetic rate, transpiration rate, and stomatal conductance than cultivar X (*p* < 0.05).

### 3.3. Effect of PNC Priming on ROS Levels in Two Rice Cultivars Under Salt Stress

**Figure 3:**
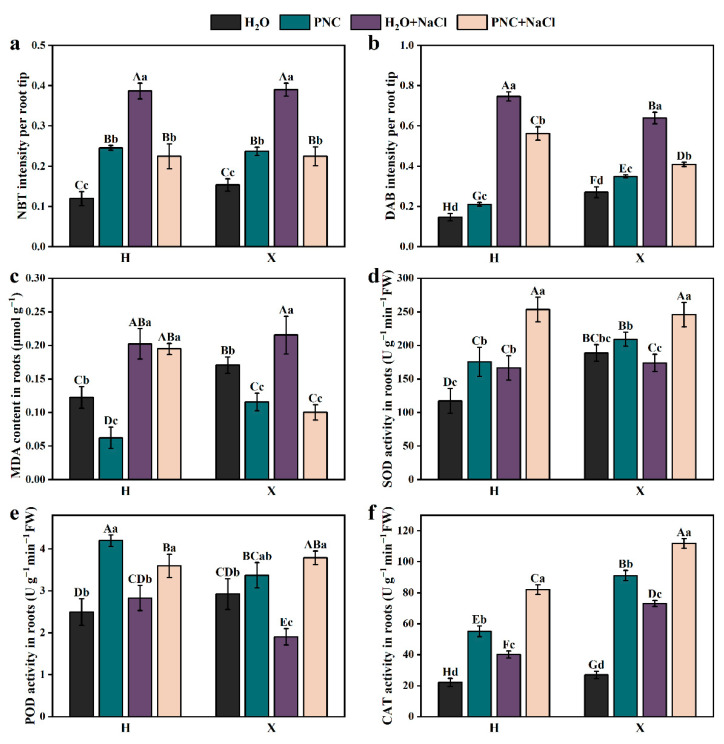
Effect of PNC priming on ROS levels in two rice cultivars under salt stress. (**a**) NBT staining intensity in rice roots; (**b**) DAB staining intensity in rice roots; (**c**) MDA content in rice roots; (**d**) SOD activity in rice roots; (**e**) POD activity in rice roots; and (**f**) CAT activity in rice roots. Columns marked with different lowercase letters indicate significant differences among treatments within the same cultivars, while different uppercase letters denote significant differences between treatments of different cultivars, based on the Duncan test (*p* < 0.05, *n* = 3).

ROS include superoxide anions (O_2_^.−^) and H_2_O_2_; excessive ROS can damage cells. Rice roots were stained by NBT and DAB (Figure S2). ImageJ-Fijj (1.46r; Java 1.6.0_20) analysis of the staining intensity (Figure 3a,b) revealed that under salt stress, the staining intensities of O_2_^.−^ and H_2_O_2_ in the roots of rice cultivars H and X were significantly higher than in normal conditions (*p* < 0.05). After PNC priming, the staining intensity of O_2_^.−^ and H_2_O_2_ decreased by 41.97–42.40 and 24.63–36.00%, respectively (Figure 3a,b). Additionally, under salt stress, the MDA content in the roots of the H and X cultivars were 1.65 and 1.26 times that of normal conditions, respectively. PNC priming reduced the MDA content, but the MDA content in cultivar H was still 1.95 times that of cultivar X (Figure 3c). These results indicate that under salt stress, PNC reduced the accumulation of O_2_^.−^, H_2_O_2_, and MDA in the rice roots, helping to maintain relative homeostasis.

To further investigate the differential effects of PNC priming on ROS scavenging in the roots of H and X cultivars under salt stress, the activity of the antioxidant enzyme was measured. The results showed that PNC priming increased the SOD, POD, CAT activity in the roots of both cultivars under normal and salt-stress conditions (Figure 3d–f). However, under salt stress, the activities of POD and CAT in the X cultivar after PNC priming were higher than in the H cultivar by 5.29 and 36.07%, respectively (Figure 3e,f). This suggests that PNC can enhance the antioxidant capacity of rice roots, but there are differences between cultivars.

### 3.4. Effect of PNC Priming on Endogenous Hormones of Two Rice Cultivars Under Salt Stress

**Figure 4:**
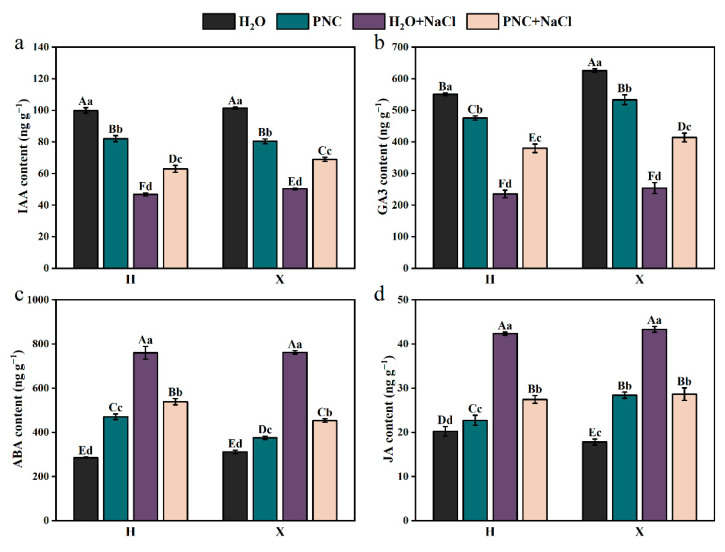
Effect of PNC priming on hormones in two rice cultivars under salt stress. (**a**) IAA content in rice roots; (**b**) GA3 content in rice roots; (**c**) ABA content in rice roots; and (**d**) JA content in rice roots. Columns marked with different lowercase letters indicate significant differences among treatments within the same cultivars, while different uppercase letters denote significant differences between treatments of different cultivars, based on the Duncan test (*p* < 0.05, *n* = 3).

Under salt stress, the level of IAA and GA3 in both rice cultivars H and X were significantly lower than in normal conditions. However, PNC priming increased IAA and GA3 levels compared with water priming under salt stress, and the enhancement effect was better for the X than the H cultivar (Figure 4a,b). For ABA and JA, their contents in both cultivars were higher under salt stress than in normal conditions, but PNC priming reduced the contents, with differences observed between the cultivars (Figure 4c,d). Notably, ABA content in cultivar X decreased by 15.82% compared to cultivar H. These results indicated that PNC enhances rice salt tolerance through stomatal closure, regulating resistance gene expression, and promoting cell elongation and division, but its effects vary between different cultivars.

### 3.5. Principal Component Analysis of Phenotypic and Physiological Indices in Two Rice Cultivars

**Figure 5:**
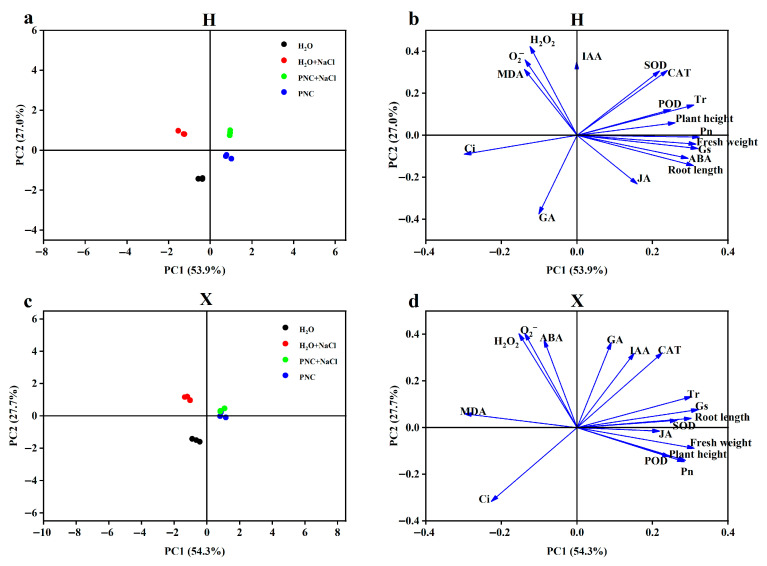
PCA of phenotypic and physiological indices in two rice cultivars. (**a**) PCA score distribution of indicators under different treatments in the H cultivar; (**b**) PCA loading plot of the H cultivar; (**c**) PCA score distribution of indicators under different treatments in the X cultivar; and (**d**) PCA loading plot of the X cultivar.

Correlations between phenotypes and various physiological indices of different salt-tolerant rice cultivars under salt stress by PNC priming were assessed using principal component analysis (PCA). PC1 and PC2 denoted the first and second principal components, respectively. Explaining 53.9% and 27.0% of the variance in the H cultivar, and 54.3% and 27.7% of the variance in the X cultivar. In the score plot (Figure 5a,c), PNC and PNC + NaCl treatments were closely related in both H and X cultivars, and PNC priming alleviated salt stress more effectively in X. The loading plots (Figure 5b,d) showed that there was a positive correlation between phenotypes, antioxidant enzymes, and photosynthetic parameters (net photosynthesis, transpiration, and stomatal conductance) in both H and X cultivars, and that there was a negative correlation between antioxidant enzymes and ROS. However, in the H cultivar, phenotypes were positively correlated with ABA and negatively with GA3; the opposite was true in the X cultivar.

### 3.6. Transcriptomic Analysis of Two Rice Cultivars Under Salt Stress Triggered by PNC

**Figure 6:**
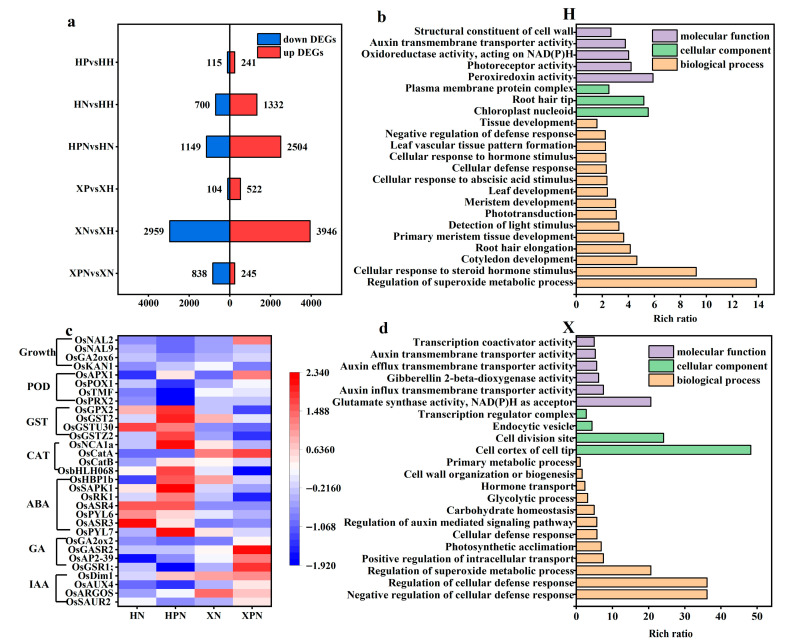
Transcriptomic analysis in two rice cultivars induced by PNC under salt stress. (**a**) The number of DEGs in the comparison groups; (**b**) GO enrichment analysis of DEGs in the H cultivar between HPN and HN; (**c**) heatmap of hormones, antioxidant enzymes, and rice growth-related genes in both cultivars under salt stress; (**d**) GO enrichment analysis of DEGs in the X cultivar between XPN and XN. HH: water priming in the H cultivar; HP: PNC priming in the H cultivar; HN: water priming + 50 mM NaCl in the H cultivar; HPN: PNC priming + 50 mM NaCl in the H cultivar; XH: water priming in the X cultivar; XP: PNC priming in the X cultivar; XN: water priming + 50 mM NaCl in the X cultivar; XPN: PNC priming + 50 mM NaCl in the X cultivar.

To explore the molecular mechanisms underlying the differential salt tolerance induced by PNC priming in two rice cultivars under salt stress, transcriptomic analysis was performed. Under normal conditions, compared to water priming, PNC priming resulted in 241 and 522 up-regulated DEGs and 115 and 104 down-regulated DEGs in the H and X cultivars, respectively. Under salt stress, PNC-primed cultivars H and X had 3653 (2504 up-regulated, 1149 down-regulated) and 1083 (245 up-regulated, 838 down-regulated) DEGs (Figure 6a), respectively, compared with water priming. This indicates that different salt-tolerant rice cultivars induced by PNC differ significantly at the transcriptome level, and the DEGs in the H cultivar were significantly higher than in the X cultivar.

In order to understand the biological functions of DEGs, we conducted GO enrichment analysis on those initiated by water or PNC under salt stress. The results revealed that in the H cultivar, PNC priming significantly enriched terms related to superoxide metabolism regulation, tissues (roots, leaves) growth and development, and cellular response to steroid hormone stimulus, compared to water priming (Figure 6b). In the X cultivar, the significantly enriched term mainly concentrated in cellular defense, superoxide metabolism, and hormone transport (IAA, GA) (Figure 6d). This indicates that the biological functions of DEGs vary between the two rice cultivars under salt stress.

We analyzed the expression of hormones (IAA, GA, ABA), antioxidant enzymes (CAT, POD, GST), and growth-related genes in both cultivars under salt stress (Figure 6c). The results showed that IAA (*OsSAUR2*, *OsARGOS*, *OsAUX4*, *OsDim1*), GA (*OsGSR1;*, *OsAP2-39*, *OsGASR2*, *OsGA2ox2*), CAT (*OsCatA*), and POD (*OsPRX2*, *OsTMF*, *OsPOX1*, *OsAPX1*) were expressed higher in the X than the H cultivar initiated by PNC in the rice roots. Conversely, ABA (*OsPYL7*, *OsASR3*, *OsPYL6*, *OsASR4*, *OsRK1*, *OsSAPK1*, *OsHBP1b*), GST (*OsGSTZ2*, *OsGSTU30*, *OsGST2*, *OsGPX2*), and *OsKAN1* which regulates plant height, were more highly expressed in the H than the X cultivar. In addition, under salt stress, compared with water priming, PNC priming was screened for 1229 and 471 DEGs belonging to transcription factors (TFs) in the cultivars H and X, respectively. Notably, the FAR1 family, which regulates light-signal transduction, was exclusively expressed in the cultivar H. The NAC and MYB_related families were expressed in the H cultivar more than the X cultivar (Figure S3).

### 3.7. Metabolomic Analysis of Two Rice Cultivars Under Salt Stress Induced by PNC

Untargeted metabolomics analysis was conducted to understand the metabolic network differences in PNC-primed salt-tolerant rice cultivars under salt stress. A total of 842 metabolites were detected. DEMs were identified using the criteria of |log_2_FC| ≥ 1, *p*-value < 0.05, and VIP value ≥ 1. Under salt stress, compared to water priming, PNC priming resulted in 77 DEMs (42 up-regulated, 35 down-regulated) in the H cultivar and 105 DEMs (68 up-regulated, 37 down-regulated) in the X cultivar (Figure S4).

**Figure 7:**
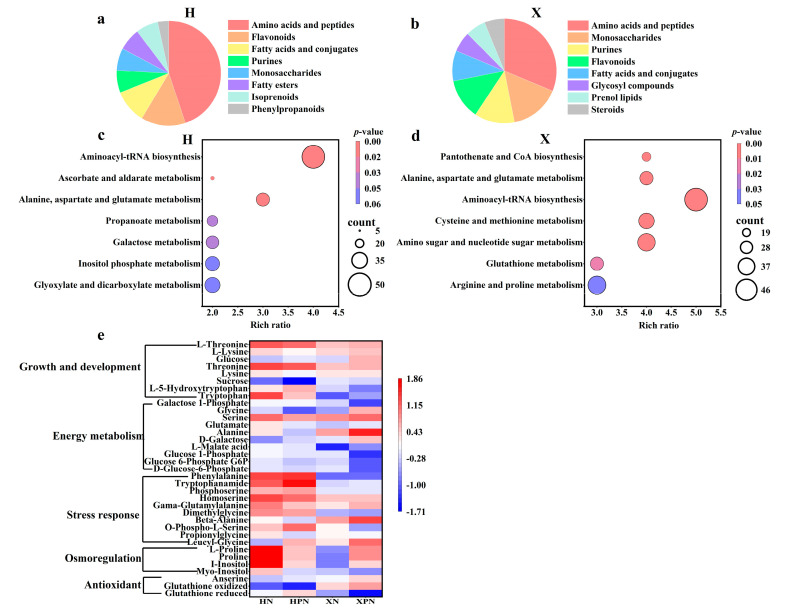
Metabolomic analysis of rice roots under salt stress induced by PNC priming. (**a**) Percentage classification of DEMs in the H cultivar between HPN and HN; (**b**) percentage classification of DEMs in the X cultivar between XPN and XN; (**c**) KEGG enrichment analysis of DEMs in the H cultivar between HPN and HN; (**d**) KEGG enrichment analysis of DEMs in the X cultivar between XPN and XN; (**e**) heatmap of antioxidants, osmoregulatory substances, energy metabolites and growth-related DEMs in both rice cultivars under salt stress. HN: water priming + 50 mM NaCl in the H cultivar; HPN: PNC priming + 50 mM NaCl in the H cultivar; XN: water priming + 50 mM NaCl in the X cultivar; XPN: PNC priming + 50 mM NaCl in the X cultivar.

DEMs were classified via the HMDB, revealing both similarities and differences among the DEMs of different cultivars. Under salt stress, compared to water priming, the top three metabolites in PNC priming were amino acids and peptides, flavonoids, and fatty acids and conjugates in the H cultivar (Figure 7a), whereas in the X cultivar, the top three metabolites were amino acids and peptides, monosaccharides, and purines/flavonoids (Figure 7b). For deeper insights into DEMs’ biological implications, KEGG enrichment analysis was performed on DEMs from PNC-primed versus water-primed groups under salt stress. The results indicated that the H cultivar’s differential metabolic pathways were mainly enriched in aminoacyl-tRNA biosynthesis, ascorbate and aldarate metabolism, alanine, aspartate, and glutamate metabolism (Figure 7c). In contrast, the X cultivar showed significant enrichment in pantothenate and CoA biosynthesis, amino sugar and nucleotide sugar metabolism, glutathione metabolism, and arginine and proline metabolism (Figure 7d).

PNC enhances plant salt tolerance by modulating antioxidants, osmoregulatory substances, and energy metabolism, but its effects vary among cultivars. In this study, compared to the H cultivar, PNC priming increased the levels of antioxidants (glutathione oxidized, anserine), osmoregulatory substances (proline), and energy metabolism (glycine, serine, alanine, D-galactose) in the X cultivar. Conversely, in the cultivar H, metabolites linked to adversity response (O-phospho-L-serine, dimethylglycine, homoserine, phosphoserine, tryptophanamide, phenylalanine) and growth/development (tryptophan, L-5-hydroxytryptophan, threonine, L-threonine) were elevated compared to the X cultivar (Figure 7e).

### 3.8. Correlation Analysis Between Phenotypic and Omics in Two Rice Cultivars

**Figure 8:**
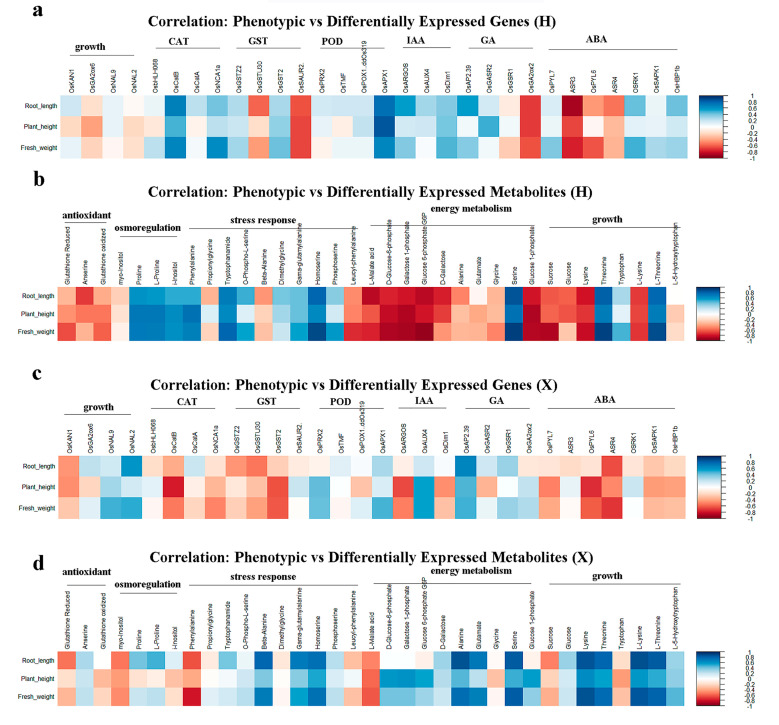
Correlation analysis between phenotypic traits and omics profiles in two rice cultivars. (**a**) Correlation analysis between phenotypic traits and differentially expressed genes in the H cultivar; (**b**) correlation analysis between phenotypic traits and differentially expressed metabolites in the H cultivar; (**c**) correlation analysis between phenotypic traits and differentially expressed genes in the X cultivar; and (**d**) correlation analysis between phenotypic traits and differentially expressed metabolites in the X cultivar.

The correlation between PNC-induced phenotypic traits and differentially expressed genes or metabolites in two rice cultivars under salt stress were evaluated. The results showed that in the H cultivar, plant height, root length, and fresh weight were positively correlated with the majority of genes involved in the CAT, IAA, and ABA pathways (Figure 8a) and with metabolites related to osmoregulation and stress response, but negatively correlated with those associated with antioxidant and energy metabolism (Figure 8b). For the X variety, the same phenotypic parameters were negatively correlated with genes linked to CAT and ABA (Figure 8c) yet positively correlated with metabolites involved in energy metabolism and growth promotion (Figure 8d).

## 4. Discussion

**Figure 9:**
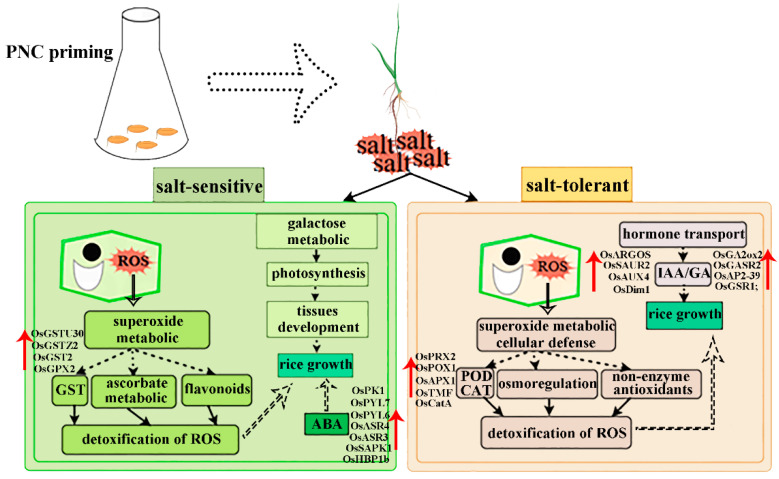
Potential mechanistic analysis of salt-tolerance differences in two rice cultivars induced by PNC.

Nanomaterials are defined as materials with at least one dimension in the nanoscale range (1–100 nm) in three-dimensional space. Exhibit unique size and surface effects that enhance plant growth, nutrient utilization, and stress resistance [25,26,27]. Among these, CeO_2_NPs demonstrates exceptional redox activity due to the coexistence of Ce^3+^/Ce^4+^ valence states on their surface [8]. Accumulating evidence indicates that CeO_2_NPs effectively activates antioxidant defense systems, scavenging excess H_2_O_2_ and O_2_^.−^ in plants [14,15,28,29]. Our study also showed that PNC increased antioxidant enzyme activities and decreased H_2_O_2_ and O_2_^.−^ content in rice roots under salt stress (Figure 3a,b), and activated pathways related to antioxidants such as glutathione, ascorbate, and aldarate metabolism (Figure 7c,d). This suggests that CeO_2_NPs can serve as the first line of defense against oxidative stress in plants and can effectively mitigate stress-induced oxidative damage [30]. However, differential responses and adaptation mechanisms to PNC exist among rice cultivars (Figure 9). The sensitive cultivar H scavenged ROS by up-regulating GST expression and activating the ascorbate/aldarate metabolism pathways (Figure 6c and Figure 7c). The salt-tolerant cultivar X showed higher CAT/POD activities and expression levels of (*OsCatA*, *OsPRX2*, *OsTMF*, *OsPOX1*, *OsAPX1*), antioxidant metabolites (glutathione oxidized, anserine), and osmoregulatory substances (proline) than the cultivar H after PNC priming (Figure 3e,f, Figure 6c and Figure 7e). Furthermore, the glutathione metabolism, arginine, and proline metabolism pathways were significantly enriched in the X cultivar (Figure 7d), showing superior ROS scavenging. This disparity may stem not only from PNC’s redox properties but also from the inherently stronger antioxidant capacity of the salt-tolerant cultivars, as evidenced by previous studies showing higher ROS scavenging in the salt-tolerant compared to salt-sensitive cultivars [28].

Photosynthesis is considered one of the most sensitive physiological indicators in response to salt stress [31], during which stomatal closure reduces CO_2_ uptake, lowering photosynthetic and transpiration rates [32]. CeO_2_NPs has been shown to enhance photosynthesis and growth in plants like brassica napus [33], spearmint [34], and alfalfa [29] under salt stress. This study also found that rice plants primed with PNC under salt stress had greater plant height, root length, and photosynthesis than those with water priming (Figure 1a,b and Figure 2). Notably, PNC was more effective in enhancing photosynthesis in the H cultivar than the X cultivar, possibly due to the significant enrichment of galactose metabolism in the H cultivar (Figure 7d). Galactose metabolism can improve photosynthesis by influencing glycolipid synthesis and the transport of photosynthetic products [35]. Additionally, the PNC-primed H cultivar showed better phenotypic improvements and higher levels of growth-related metabolites (tryptophan, L-5-hydroxytryptophan, and threonine) than the X cultivar (Figure 1 and Figure 7e), which may result from photosynthesis providing ample materials and energy for growth.

Phytohormones are crucial for both plant growth/development and responses to biotic/abiotic stresses [36,37]. For instance, ABA enhanced root-system architecture and stress resilience [38,39]. In addition, phytohormones contribute greatly to the signal sensing and defense system of stress [40]. Under stress conditions, the synthesis of growth-promoting hormones decreases, while that of stress-related hormones increases [41,42]. For example, the expression of most DEGs associated with ABA biosynthesis was significantly up-regulated in populus under salt stress [43]. Similarly, in this study, salt stress reduced IAA/GA3 levels but increased ABA/JA levels in rice roots (Figure 4). CeO_2_NPs can activate hormone signal transduction and regulate hormone levels [44], which was confirmed in this study (Figure 4). However, differences existed between rice cultivars. The tolerant X cultivar had higher IAA/GA3 levels and a higher expression of related signal-response genes (*OsARGOS*/*OsGASR2*), while the sensitive cultivar H showed higher ABA levels and expression of ABA-related genes, including receptor proteins (*OsPYL7*, *OsPYL6*), transcription factors (*OsHBP1b*), and signaling kinases (*OSRK1*, *OsSAPK1*) (Figure 4 and Figure 6). This may be due to the fact that the sensitive cultivars themselves are weakly tolerant to salt and remain more sensitive to stress response even after pre-treatment with PNC, but the exact reason for this remains to be further investigated.

## 5. Conclusions

PNC priming enhanced rice salt tolerance, but the mechanisms vary between cultivars. In the salt-sensitive cultivar H, PNC up-regulated GST expression and activated the ascorbate/aldarate metabolism pathway to maintain redox balance, while also activating stress-response signaling, and boosting photosynthesis to improve salt tolerance. The salt-tolerant cultivar X enhanced salt tolerance by activating cellular defense responses, elevating CAT/POD activities, and enhancing proline and glutathione metabolism to improve antioxidant capacity, along with increased IAA/GA3 levels and up-regulated expression of related signaling genes (*OsARGOS*/*OsGASR2*). This study advances our understanding of how nanomaterials like PNC can be harnessed to improve rice salt tolerance, and contribute to the broader field of nanotechnology in agriculture.

## Data Availability

The original contributions presented in this study are included in the article/Supplementary Materials. Further inquiries can be directed to the corresponding author.

## Supplementary Materials

The following supporting information can be downloaded at: https://www.mdpi.com/article/10.3390/antiox14080994/s1, Figure S1. Effects of different PNC concentrations on rice growth under salt stress. (a) Plant height after 7-day treatment; (b) root length after 7-day treatment. Columns marked with different lowercase letters indicate significant differences among PNC treatment concentrations at the same NaCl concentration, based on the Duncan test (*p* < 0.05, *n* = 5). Figure S2. Effect of PNC priming on NBT and DAB staining images in two rice cultivars under salt stress. (a) An image of the root system of the H cultivar under NBT staining; (b) an image of the root system of the X cultivar under NBT staining; (c) an image of the root system of the H cultivar under DAB staining; (d) an image of the root system of the X cultivar under DAB staining. Figure S3. Transcriptomic analysis in two rice cultivars induced by PNC under salt stress. (a) TFs analysis of DEGs in the H cultivar between HPN and HN; (b) TFs analysis of DEGs in the X cultivar between XPN and XN. Figure S4. Metabolomic analysis of rice roots under salt stress induced by PNC priming. (a) The number of up-regulated/down-regulated metabolites between HPN and HN; (b) the number of up-regulated/down-regulated metabolites between XPN and XN.
