# Supplementary material for: An Analysis of the Different Salt-Tolerance Mechanisms in Rice Cultivars Induced by Cerium Oxide Nanoparticles
Source: Antioxidants (Basel). 2025 Aug 13;14(8):994. doi: 10.3390/antiox14080994 (PMC12383031; doi:10.3390/antiox14080994)
Supplement: Supplementary file 1 [file antioxidants-14-00994-s001.zip › antioxidants-3708597-supplementary.pdf]

An Analysis of the Different Salt-Tolerance Mechanisms in Rice Cultivars Induced by Cerium Oxide Nanoparticles

Chunmei Yang [1,†], Qing Bu [1,†], Tao Su [1], Tian Wang [1], Zaid Khan [2], Mingwei Li [1], Juntian Wu [1], Xiaodan Di [1], Yong Chen [1,*] and Jing An [1,*]

[1] College of Agriculture, South China Agricultural University, Guangzhou 510642, China
[2] College of Natural Resources and Environment, South China Agricultural University, Guangzhou 510642, China
* Correspondence: chenyong@scau.edu.cn (Y.C.); anjing@scau.edu.cn (J.A.)
† These authors contributed equally to this work.

Supplementary materials

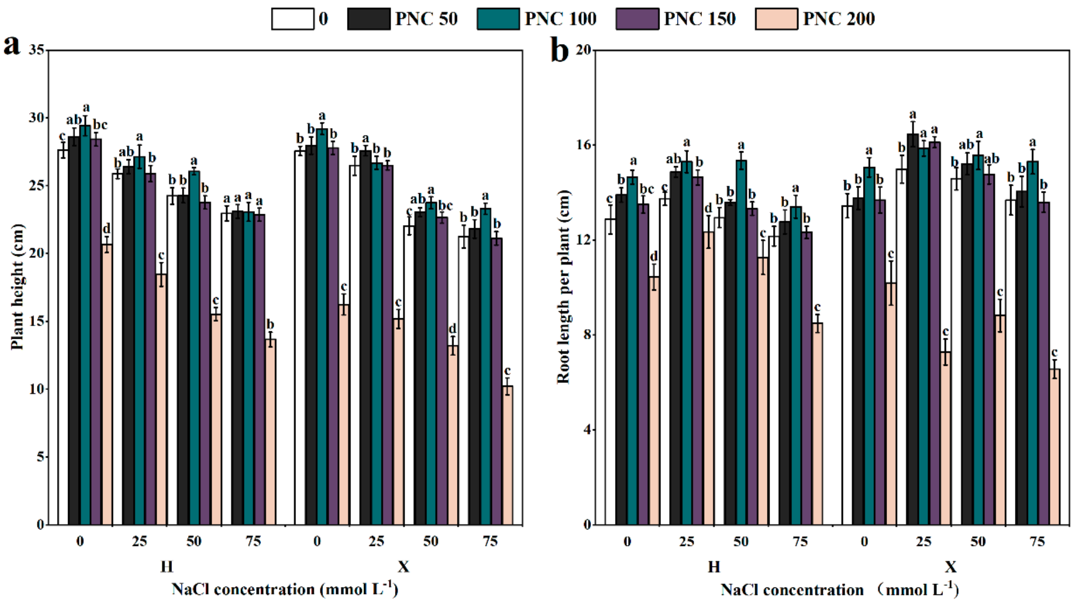

Figure S1. Effects of different PNC concentrations on rice growth under salt stress. (a) Plant height after 7-day treatment, (b) root length after 7-day treatment. Columns marked with different lowercase letters indicate significant differences among PNC treatment concentrations at the same NaCl concentration, based on the Duncan test ($p < 0.05$, n=5).

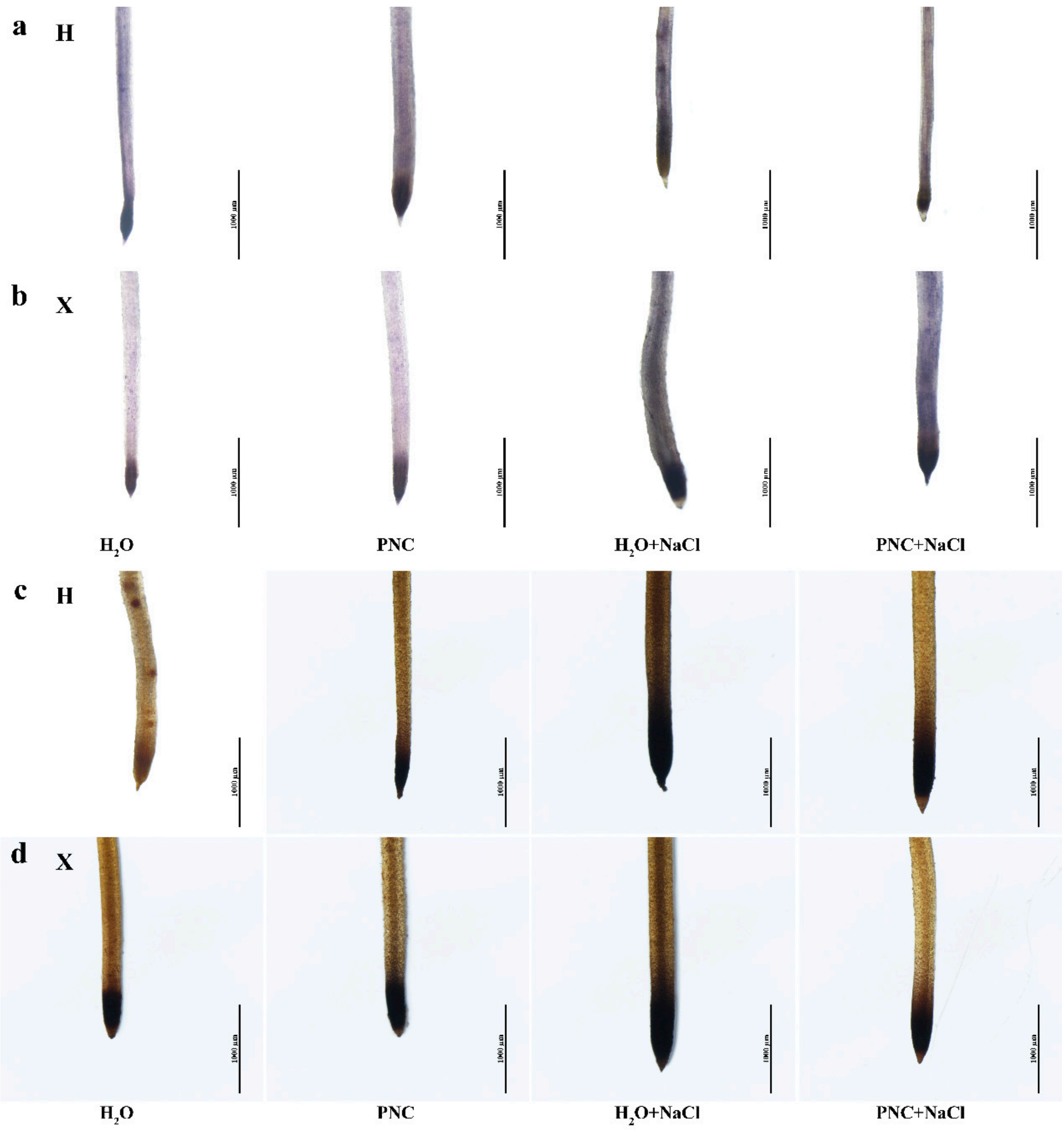

Figure S2. Effect of PNC priming on NBT and DAB staining images in two rice cultivars under salt stress. (a) Root system of H cultivar NBT staining image, (b) root system of X cultivar NBT staining image, (c) root system of H cultivar DAB staining image, (d) root system of X cultivar DAB staining image.

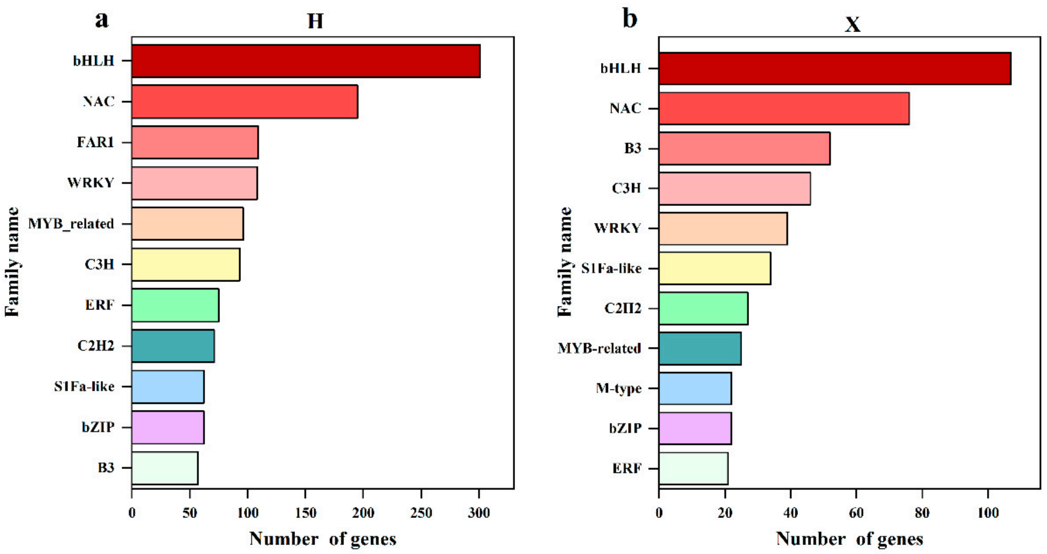

Figure S3. Transcriptomic analysis in two rice cultivars induced by PNC under salt stress. (**a**) TFs analysis of DEGs in the H cultivar between HPN and HN, (**b**) TFs analysis of DEGs in the X cultivar between XPN and XN.

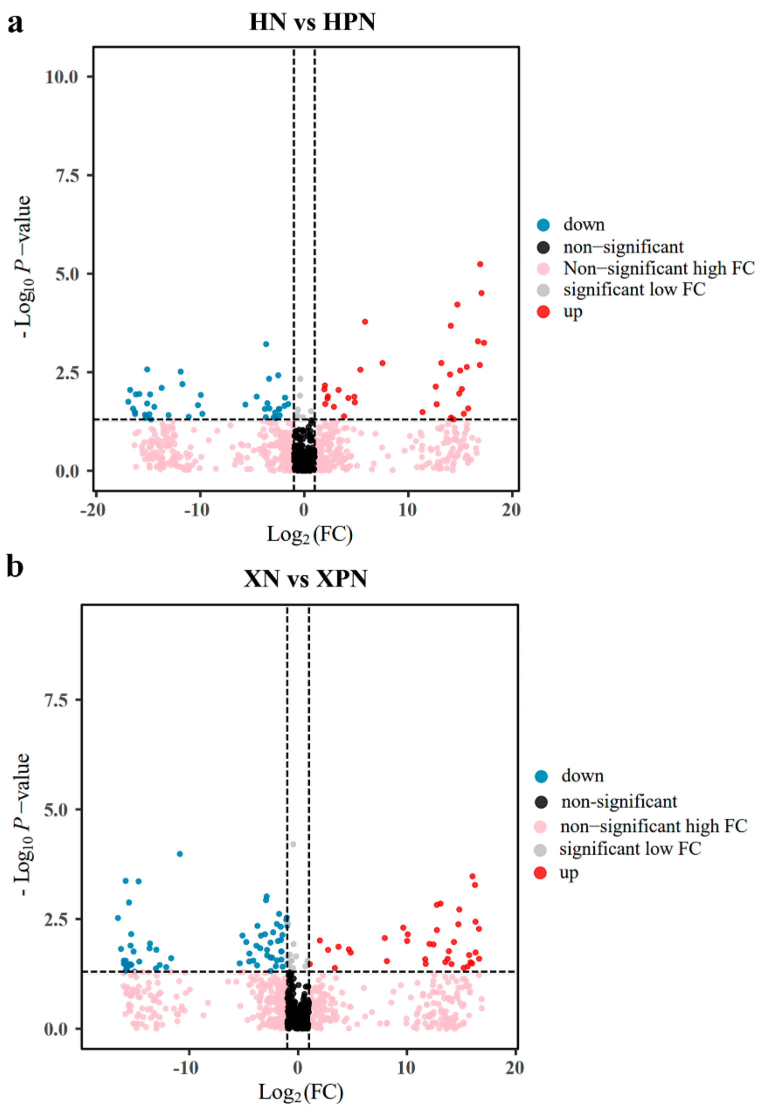

Figure S4. Metabolomic analysis of rice roots under salt stress induced by PNC priming. (**a**) The number of up-regulated/down-regulated metabolites between HPN and HN, (**b**) the number of up-regulated/down-regulated metabolites between XPN and XN.
